# Supplementary material for: Excess mortality attributable to antimicrobial-resistant bacterial bloodstream infection at a tertiary-care hospital in Indonesia
Source: PLOS Glob Public Health. 2022 Jul 20;2(7):e0000830. doi: 10.1371/journal.pgph.0000830 (PMC10021607; doi:10.1371/journal.pgph.0000830)
Supplement: S1 Table — (PDF) [file pgph.0000830.s001.pdf]

S1 Table. Pathogenic organisms isolated from blood culture among patients presenting at Wahidin Hospital, Makassar, Indonesia, from 2015 to 2018*

**Organisms**	**Neonatal** (age ≤28 days)	**Pediatric** (age >28 days and <18 years)	**Adults** (age ≥18 years)
Gram-negative bacteria			
Enterobacteriaceae			
Escherichia coli	5	21	77
Klebsiella pneumonia	48	41	82
Enterobacter cloacae	6	15	18
Serratia spp.	17	6	6
Salmonella spp.	0	10	3
Proteus mirabilis	0	2	3
Other Enterobacteriaceae**	2	2	10
Non-Enterobacteriaceae			
Acinetobacter spp.	12	39	122
Pseudomonas aeruginosa	3	19	46
Burkholderia cepacia	31	209	280
Pseudomonas spp.	1	5	13
Stenotrophomonas maltophilia	0	3	9
Sphingomonas paucimobilis	1	1	9
Burkholderia pseudomallei	0	0	9
Achromobacter spp.	1	7	3
Moraxella spp.	1	3	1
Aeromonas spp.	0	4	6
Other non-Enterobacteriaceae***	5	5	8
Unspecified Gram negatives	0	0	1
Gram-positive bacteria			
Staphylococcus aureus	4	52	191
Enterococcus spp.	1	6	53
Other Gram positives****	3	12	25
Unspecified Gram positives	0	1	1
Fungi			
Candida spp.	16	7	29
Cryptococcus spp.	1	1	5
Trichosporon asahii	0	1	0
Unspecified fungi	9	8	22
Polymicrobial infections *****	0	2	1
Overall	167	482	1,033

* Only the first isolate per patient was included in the analyses. A repeated blood culture positivity for common commensal organisms within the same admission was categorized as BSI in the study ** Other Enterobacteriaceae included *Cronobacter dublinensis* (in 0 neonatal patient, 1 pediatric patient and 0 adult patients [n=0/1/0]), *Cronobacter sakazakii* (n=0/0/1), *Enterobacter aerogenes* (n=1/0/1), *Kluyvera intermedia* (n=1/0/0), *Morganella morganii* (n=0/0/1), *Pantoea* spp (n=0/1/3), *Providencia stuartii* (n=0/0/2), *Raoultella ornithinolytica* (n=0/0/1), *Shigella spp* (n=0/0/1). *** Other non-Enterobacteriaceae

included *Brevundimonas diminuta* (n=0/0/1), *Chryseobacterium indologenes* (n=1/0/2), *Comamonas testosteroni* (n=0/1/0), *Elizabethkingia meningoseptica* (n=3/0/1), *Ochrobactrum anthropi* (n=1/2/3), *Oligella ureolytica* (n=0/1/0), *Pasteurella pneumotropica* (n=0/0/1), *Shewanella algae* (n=0/1/0). **** Other Gram positives included *Aerococcus viridans* (n=0/0/1), *Erysipelothrix rhusiopathiae* (n=0/1/0), *Gemella morbillorum* (n=0/0/1), *Granulicatella elegans* (n=0/1/1), *Leuconostoc mesenteroides* (n=1/6/8), *Pediococcus pentosaceus* (n=0/0/1), *Staphylococcus arlettae* (n=0/0/1), *Staphylococcus haemolyticus* (n=0/1/1), *Staphylococcus hominis* (n=1/0/3), *Staphylococcus intermedius* (n=0/1/0), *Staphylococcus pseudintermedius* (n=1/0/0), *Staphylococcus* spp. (n=0/1/4), *Streptococcus dysgalactiae* (n=0/0/2), *Streptococcus pyogenes* (n=0/1/2). ***** Polymicrobial infections included *Klebsiella pneumonia* and *Pseudomonas aeruginosa* (n=0/0/1), *Acinetobacter* spp. and *Stenotrophomonas maltophilia* (n=0/1/0), *Escherichia coli* and *Salmonella* spp. (n=0/1/0).
