# Supplementary material for: Excess mortality attributable to antimicrobial-resistant bacterial bloodstream infection at a tertiary-care hospital in Indonesia
Source: PLOS Glob Public Health. 2022 Jul 20;2(7):e0000830. doi: 10.1371/journal.pgph.0000830 (PMC10021607; doi:10.1371/journal.pgph.0000830)
Supplement: S2 Table — (PDF) [file pgph.0000830.s002.pdf]

S2 Table. Proportions of patients with blood cultures positive for antibiotic-resistant isolates of the five targeted pathogens

Pathogens	Total	Community origin	Hospital origin	P value
Escherichia coli				
3rd generation cephalosporin-resistant	78% (81/104)	50% (6/12)	82% (75/92)	0.01
3rd generation cephalosporin-resistant plus carbapenem resistant	4% (4/104)	0% (0/12)	4% (4/92)	0.46
Klebsiella pneumonia				
3rd generation cephalosporin-resistant	56% (96/171)	56% (18/32)	56% (78/139)	0.99
3rd generation cephalosporin-resistant plus carbapenem resistant	25% (43/171)	25% (8/32)	25% (35/139)	0.98
Staphylococcus aureus				
Methicillin-resistant	51% (124/245)	61% (30/49)	48% (94/196)	0.10
Acinetobacter spp.				
Carbapenem-resistant	48% (82/171)	13% (1/8)	50% (81/163)	0.04
Pseudomonas aeruginosa				
Carbapenem-resistant	19% (13/68)	33% (2/6)	18% (11/62)	0.35
