# Supplementary material for: Excess mortality attributable to antimicrobial-resistant bacterial bloodstream infection at a tertiary-care hospital in Indonesia
Source: PLOS Glob Public Health. 2022 Jul 20;2(7):e0000830. doi: 10.1371/journal.pgph.0000830 (PMC10021607; doi:10.1371/journal.pgph.0000830)
Supplement: Attachment — Submitted filename: Response to Reviewers.docx [file pgph.0000830.s004.docx]

**Re:** PGPH-D-22-00421

Epidemiology and excess mortality attributable to antimicrobial-resistant bacterial infection at a tertiary-care hospital in Indonesia.

PLOS Global Public Health

Dear Editor,

Thank you for your e-mail concerning the above manuscript. We are grateful for the helpful comments made by the reviewers and have addressed them as detailed below.

COMMENTS TO THE JOURNAL EDITOR:

Journal Requirements:

1. Please amend your detailed Financial Disclosure statement. This is published with the article. It must therefore be completed in full sentences and contain the exact wording you wish to be published.

- State the initials, alongside each funding source, of each author to receive each grant.

***Answer****: The study was funded by the Wellcome Trust (219651/Z/19/Z). For the purpose of Open Access, the author has applied a CC BY public copyright license to any Author Accepted Manuscript version arising from this submission. The funders of the study had no role in study design, data collection and analysis, decision to publish, or writing of the report.*

2. We do not publish any copyright or trademark symbols that usually accompany proprietary names, eg (R), (C), or TM (e.g. next to drug or reagent names). Please remove all instances of trademark/copyright symbols throughout the text, including VITEK®2 on page 6.

***Answer****: All instances of trademark/copyright symbols have been removed as suggested.*

COMMENTS TO THE AUTHOR:

Reviewer #1: In table 2, how can the authors calculate the mortality of outpatients?

In table 3, those factors were demography characterized, not epidemiology? There were two aims of this study - epidemiology, and mortality attributable, however, the authors have just described mortality attributable, not mention the epidemiology.

***Answer****: In Table 2, the term ‘outpatient’ represents ‘patients admitted from outpatient department’, and the mortality presented was in-hospital mortality. The term ‘type of admission’ has been revised to ‘Admission from’ in Table 1 and 2 for clarity. The term ‘epidemiology’ in the title has been removed. The term ‘epidemiology’ in the introduction and conclusion has been revised to ‘proportion, rate’ for clarity.*

Reviewer #2: In their manuscript entitled, “Epidemiology and excess mortality attributable to antimicrobial-resistant bacterial infection at a tertiary-care hospital in Indonesia,” Dr. Tauran, and colleagues, utilize routinely collected admission and microbiology data from a tertiary hospital in Indonesia to explore attributable mortality of drug-resistant bloodstream infections occurring in hospitalized patients over a 3-year period (2015-18). The manuscript is well written, comprehensive in its approach and aligns well with efforts underway to address the problem of mortality due to AMR infections globally. The analytic approach to understanding excess mortality and deaths was clearly described and can be reproduced in other similar settings. Some clarity is required in terms of potential areas of bias introduced by the limitations in the datasets. The following comments are included to help address some of these minor issues:

***Answer****: We are grateful for the positive review.*

Title: Given that the study includes only blood-stream infections, the title should explicitly state that the infections described in this study are AMR bacterial blood-stream infections rather than generally as ‘infection’.

***Answer****: The title has been revised to “Excess mortality attributable to antimicrobial-resistant bacterial bloodstream infections at a tertiary-care hospital in Indonesia.” as suggested.*

Introduction:

 Line 57: The sentence starting, “The 4.95 million deaths…” is difficult to digest and would benefit from rephrasing for clarity, perhaps alluding to similar language to describe the replacement and additive scenarios later in the manuscript.

***Answer****: The section has been revised for clarity as follows, “The 4.95 million deaths were estimated using a predictive statistical modelling and based on a counterfactual scenario of no infection (i.e. a scenario that if AMR infections would have been prevented, patients would not have experienced an infection) (1). Alternatively, 1.27 million deaths were also estimated based on an alternative counterfactual scenario of antimicrobial-susceptible (AMS) infections (i.e. a scenario that if AMR infections would have been prevented, AMR infections would have been replaced by AMS infections) (1).”*

 Line 70: Rephrase for clarity, perhaps by deleting ‘which’ so that ‘reported’ can serve as the verb in the sentence.

***Answer****: The word “which” in line 70 has been deleted as suggested.*

 Line 75: the definition for ‘incidence rates’ in parentheses does not actually define a ‘rate’. Suggest removing the word rate and leaving as ‘incidence’ or ‘cumulative incidence’ throughout the text.

***Answer****: The term ‘incidence’ and ‘incidence rate’ has been changed to ‘rate’ as per WHO GLASS.*

Definitions:

 Lined 135-6: Is it possible that patients included in Cohort 3 had a diagnosis for an infection caused by an AMR bacteria isolated from a compartment other than the bloodstream? If yes, then a proportion of these patients might still have a drug-resistant infection, just not a BSI. Ideally, it would be useful to understand the difference in outcome for patients with drug resistant infection that are BSI versus not BSI as a separate cohort. Were these data reported as per GLASS requirements? It is important to highlight this distinction here and elsewhere in the document (see comment above re: Title), including the limitations section in the Discussion.

***Answer****: The WHO GLASS method for estimating attributable mortality of AMR BSI does not mention these data (comparing BSI vs. not BSI; and comparing AMR BSI vs. AMR infection but not BSI). The limitation has been revised as follow, “Third, a small proportion of patients with AMS BSI (Cohort 2) and patients with no infection (Cohort 3) included in the analysis might have AMR infection in other organs (such as pneumonia or urinary tract infections).” Title has been revised as suggested.*

Data analysis:

 Lines 168-9: The sentence states that you are comparing mortality risk between patients with AMR BSI and patients without infection. Yet, the parentheses after ‘non-infection’ include Cohorts 1 and 2. Given that Cohorts 1 and 2 comprise patients with infection, further explanation is required to explain how Cohorts 1 and 2 can be used to represent patients without infection. Is it because patients who have yet to have a BSI identified by blood culture within cohorts 1 and are not yet classified as having infection? If this is the case, please also explain to what extent this approach introduces bias.

***Answer****: This approach was used to reduce the immortal time bias as illustrated in Reference 13 (The WHO GLASS method for estimating attributable mortality of AMR BSI). The following sentences have been added to the discussion for clarity, “Third, we used exposure density sampling as recommended by the WHO GLASS (13), which means that for every patient with an AMR BSI by a target pathogen, an unexposed patient is matched based on duration of hospital stay before infection, on an individual level. This approach can reduce immortal time bias, which is an artificial survival advantage among patients with hospital-acquired infections who had to survive long enough to be able to develop the infection. If the exposure density sampling was not used, the impact of AMR BSI could be underestimated (13).”*

Results:

 Line 223: I think the authors did not mean to include ‘Of’ at the beginning of the sentence. Please delete.

***Answer****: The word “of” has been deleted as suggested.*

 Lines 223-6: The sentence states that the most common observed pathogens were B. cepacia, S. aureus, Acinetobacter spp and K. pneumoniae. These represent a total of 1,113 (66.2%) of the total 1682 isolated pathogenic organisms described in the S1 Table. The paragraph, however, refers to 8341 patients with one episode of BSI. Please reconcile these numbers.

***Answer****: The typo has been corrected. The sentences have been revised into “Overall, 1,682 patients had at least one episode of BSI during the study period (S1 Table). The most common pathogen observed were B. cepacia (n=520; 30.9%), S. aureus (n=247; 14.7%), followed by Acinetobacter spp. (n=174; 10.3%) and K. pneumoniae (n=172: 10.2%) (S1 Table). Three patients had polymicrobial infections.”*

 Lines 230-235: This information is included verbatim in the caption for Figure 1. Is it possible to remove it in one of the places to avoid redundancy?

***Answer****: The caption in Figure 1 has been revised to avoid redundancy.*

Discussion:

 Lines 387-89:

o Given the low utilization of blood cultures overall, do the authors have any data to evaluate the potential association between illness severity and clinician threshold for obtaining a blood culture (i.e., to ascertain level of selection bias)? For instance, if clinicians are more likely to order a blood culture when a patient is critically ill, then the cohorts comprising patients with AMR and AMS infections would also reflect the sickest patients and would explain why there is a significant difference in mortality between those patients and the patients in cohort 3.

***Answer****: To our knowledge, there have been no studies evaluating the bias to excess mortality and deaths attributable to AMR BSI caused by low blood culture utilization rate. The limitation has been revised to included a possibility on under-estimation of excess deaths attributable to AMR BSI as follows, “It is still unclear how a low blood culture utilization rate or delayed blood culture could impact the comparison of mortality between patients with AMR BSI and AMS BSI, and between patients with AMR BSI and non-infection (29). Nonetheless, we hypothesized that a number of patients with AMR BSI died without being diagnosed because of the low blood culture utilization rate; therefore, excess deaths attributable to AMR BSI was likely underestimated.”*

o Also, do the authors know what proportion of cohort 3 patients had blood cultures but were negative? If there was a sizeable proportion of such patients, presenting these data explicitly could help allay the above concern about selection bias.

***Answer****: The following sentence has been added for clarity, “In the cohort 3, most patients (90.2%, n=69,411/76,987) had no blood culture taken, 8.6% of patients (6,659/76,897) had blood culture taken and culture negative for pathogens, and 1.2% of patients (917/76,897) had blood culture positive for a non-target pathogen.”*

*All contributing authors have reviewed and concurred with the revised manuscript.*

*Kind regards,*

*Patricia M. Tauran*
